# Comparison of two techniques in proximal anastomosis in acute type A aortic dissection

**DOI:** 10.3389/fcvm.2022.1047939

**Published:** 2022-10-26

**Authors:** Changcun Fang, Shan Gao, Xiao Ren, Xinyan Pang, Xin Zhao, Zengshan Ma, Chen Wang, Kai Liu

**Affiliations:** ^1^Department of Cardiovascular Surgery, Thoracoscopy Institute of Cardiac Surgery, Qilu Hospital of Shandong University, Jinan, China; ^2^Department of Anesthesia, Zhangqiu People’s Hospital, Jinan, China; ^3^Department of Extracorporeal Circulation, Wuhan Asia Heart Hospital, Wuhan, China; ^4^Department of Cardiovascular Surgery, The Second Hospital of Shandong University, Jinan, China

**Keywords:** adventitial eversion, prosthesis eversion technique, proximal anastomosis, acute type A aortic dissection (AAAD), open surgery

## Abstract

**Background:**

The proximal anastomosis is an important procedure during the acute type A aortic dissection (AAAD) surgery. The conventional method is a double patch sandwich technique with Teflon felt. Adventitial eversion and prosthesis eversion technique as a novel approach has been applied to many patients in our center. Herein, This technique would be introduced, and the perioperative and 1-year follow-up results of the two different anastomosis methods were also evaluated.

**Methods:**

Between December 2017 and May 2021, 143 AAAD patients who underwent total arch replacement (TAR) and frozen elephant trunk (FET) implantation were included in this retrospective study. Patients were divided into the eversion technique group (adventitial eversion and prosthesis eversion technique for proximal anastomosis, *n* = 64) and the sandwich technique group (*n* = 79).

**Results:**

The medical records were analyzed and compared between the groups. The mean operation time was 466 ± 73 min in the eversion technique group and 513 ± 81 min in the sandwich technique group (*P* < 0.001). Compared with the sandwich technique group, the eversion technique group also showed a shorter time on proximal anastomosis (38 ± 12 min vs. 58 ± 20 min, *P* < 0.001), cardiopulmonary bypass (195 ± 26 vs. 211 ± 40 min, *P* = 0.003), and aortic cross-clamp (120 ± 23 min vs. 134 ± 27 min, *P* = 0.002). Furthermore, a decreased proportion of >600 ml fresh frozen plasmas transfusion was observed in eversion technique group (10.9% vs. 34.2%, *P* = 0.002). No statistical differences were found in the postoperative morbidities and 1-year follow-up outcomes.

**Conclusion:**

Proximal anastomosis with adventitial eversion and prosthesis eversion technique is a promising surgical option for AAAD patients, with favorable perioperative and 1-year follow-up results.

## Introduction

Acute type A aortic dissection (AAAD) is a life-threatening disease carrying a high risk of mortality. Open surgery is the primary treatment for AAAD patients. In the past decades, great progress has been made in the methods of AAAD surgery, such as the introduction of frozen elephant trunk (FET), which has simplified the surgical procedures and decreased the mortality rate (1). At present, total arch replacement (TAR) plus FET is the most common surgical method for AAAD treatment. However, high postoperative morbidities, such as neurologic deficit, bleeding, paraplegia, and future aortic root dilation, remain challenges for the cardiac surgeons (2).

Proximal anastomosis is a crucial part during the AAAD surgery (1). As the dissected aortic stump is fragile, improper repair may lead to the anastomosis disintegration and uncontrollable bleeding. The double patch sandwich technique with Teflon felt is the conventional surgical procedure for proximal anastomosis (3). However, the double patch sandwich technique still has limitations and may increase the aortic cross-clamp time, and subsequently result in the complications such as intraoperative bleeding and organ dysfunction. Herein, we tried to explore a modified approach—adventitial eversion and prosthesis eversion technique for the proximal anastomosis of AAAD for the last several years.

In this retrospective study, adventitial eversion and prosthesis eversion technique was used for proximal anastomosis of AAAD in dozens of cases. The detailed procedures were introduced, and the perioperative and 1-year follow-up outcomes compared with the traditional sandwich technique were evaluated.

## Materials and methods

### Patient selection

**FIGURE 1:**
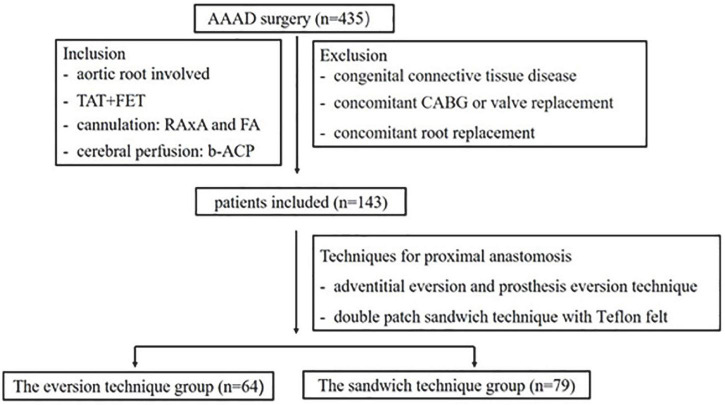
Schematic of the study cohort. Between December 2017 and May 2021, 435 patients with AAAD underwent surgical repair in our department. 143 patients were included in this study according to the inclusion and exclusion criteria. These patients were divided into two groups according to the repair methods of proximal anastomosis, the eversion technique group (adventitial eversion and prosthesis eversion technique for proximal anastomosis, *n* = 64) and the sandwich technique group (double patch sandwich technique with Teflon felt for proximal anastomosis, *n* = 79). AAAD, acute type A aortic dissection; TAT, total arch replacement; TET, frozen elephant trunk; RAA, right axillary artery; FA, femoral artery; b-ACP, bilateral antegrade cerebral perfusion; CABG, coronary artery bypass graft.

This study was conducted in accordance with the Declaration of Helsinki (as revised in 2013). The study protocol was approved by the Medical Ethics Committee of Qilu Hospital of Shandong University (Reference No. KYLL-2017KS-195). Between December 2017 and May 2021, a cohort of 435 consecutive patients with AAAD underwent surgical repair in our department. Of these patients, 143 cases were included in this study according to the inclusion and exclusion criteria (Figure 1). AAAD diagnosis was confirmed by the computerized tomographic angiography (CTA).

The inclusion criteria were: (1) the aortic root involved in the aortic dissection; (2) AAAD patients who underwent TAR and FET (TAT + FET); (3) arterial cannulation method: right axillary artery plus femoral artery; (4) bilateral antegrade cerebral perfusion (b-ACP); and (5) without other organ dysfunction prior to operation. The exclusion criteria were: (1) congenital connective tissue disease such as Marfan syndrome and (2) concomitant other procedures such as CABG, valvular replacement or valvuloplasty, or aortic root replacement. The indications of TAR + FET in our center were aortic arch dissection, aortic arch dilation (≥50 mm), and concurrent dissection in descending aorta. All the patients underwent operations within 12 h of the initial diagnosis. Both conventional sandwich technique and adventitial eversion and prosthesis eversion techniques were performed by the same surgeon and his team.

According to the methods of proximal anastomosis, patients were retrospectively divided into two groups, the eversion technique group (adventitial eversion and prosthesis eversion technique for proximal anastomosis, *n* = 64) and the sandwich technique group (double patch sandwich technique with Teflon felt for proximal anastomosis, *n* = 79). There was no particularity in the patient selection between the two groups. In other words, the severity of aortic dissection between the two groups has no difference.

### Main surgical procedures

#### Step 1: Anesthesia and CPB

All patients underwent the operation with general anesthesia and median sternotomy. Intraoperative transesophageal echocardiography probe was routinely inserted for the detection of any abnormality before weaning from CPB. Cardiopulmonary bypass was established by the cannulas in the right axillary artery and the femoral artery for perfusion, and vena cava for veinous drainage. The left atrial vent was inserted into the left atrium *via* the right superior pulmonary vein. Myocardial protection was accomplished with the antegrade infusion of cold blood cardioplegia. The ascending aorta was opened longitudinally between the brachiocephalic trunk and sinus-tubular junction (STJ).

#### Step 2: Proximal anastomosis

##### Adventitial eversion and prosthesis eversion for proximal anastomosis (the eversion technique group)

**FIGURE 2:**
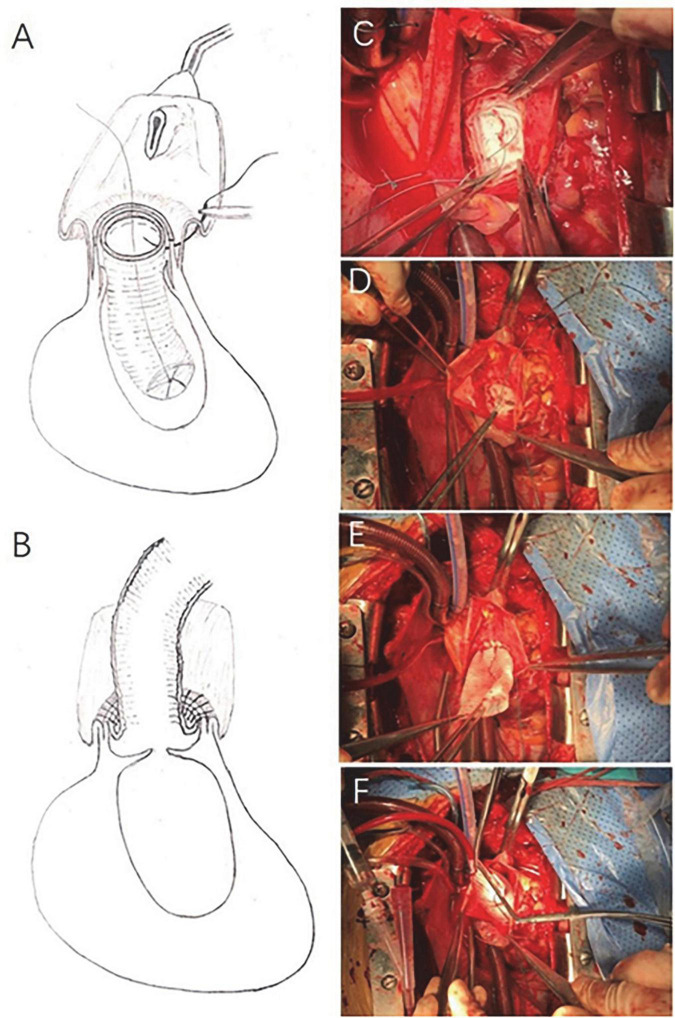
Adventitial eversion and prosthesis eversion technique for proximal anastomosis. A segment of artificial graft was inverted and inserted into the aortic root **(A,C)**. During the suture, the adventitia above the suture line was everted to form two layers of adventitia **(A).** The two layers of adventitia were directly anastomosed to the inverted part of the graft with continuous 4–0 prolene **(A)**. Thereafter, the rest part of the artificial graft was dragged out of the aortic root **(B,D,E)**. Afterward, cardioplegia was infused through the artificial graft in order to check any possible bleeding at the anastomosis site and aortic valve function **(F)**.

A segment of artificial graft was inverted and inserted into the aortic root (Figures 2A,C). During the suturing, the adventitia above the suture line was everted to form two layers of adventitia (Figure 2A). Then the two layers of adventitia were anastomosed continuously to the inverted part of the graft with 4–0 prolene (Figure 2A). Thereafter, the rest part of the artificial graft was dragged out of the aortic root (Figures 2B,D,E). Finally, cold blood cardioplegia was infused through the artificial graft with a relatively high pressure (approximate 200 mmHg measured in the infusing line) in order to check any leak in the anastomosis site and aortic valvular function (Figure 2F). If any leak was detected in the anastomosis site, additional stitches were applied to achieve hemostasis and checked with cardioplegia infusion again. Regarding aortic valvular regurgitation, initial view of the valvular structure, then cardioplegia infusion in the artificial graft with a relatively high pressure, and concomitant observation of left ventricular expansion were carried out in sequence. Once aortic valvular insufficiency was observed, valve replacement or valvuloplasty was conducted, but the case was excluded from the current study.

##### Double patch sandwich technique with Teflon felt for aortic root repair (the sandwich technique group)

Teflon felt strips were placed inside and outside the suture line, respectively. Continuous transverse mattress suture with 4–0 prolene was performed to finish the double patch sandwich. Then, a segment of artificial graft was anastomosed continuously to the double patch sandwich with 4–0 prolene. Afterward, cardioplegia was infused through the artificial graft in order to check for any leak in the anastomosis site and to test the aortic valvular function. The remained procedures were similar to the eversion technique group.

When the dissection involves part of the coronary opening and the surrounding intima of the coronary opening is intact, special reinforcement of the coronary artery is not necessary for performing these two techniques. When the surrounding intima of the coronary opening is not intact, pericardium may be used for the reinforcement of the coronary artery.

#### Step 3: TAT + FET

After the proximal anastomosis, circulatory arrest of the body lower part was performed at moderate hypothermia (Nasopharyngeal temperature 26–27^°^C). Bilateral cerebral perfusion was achieved *via* the right axillary artery and left common carotid artery (LCCA). The aortic arch was opened longitudinally, and thereafter the stent elephant trunk was placed into the true lumen of the descending aorta. The orifice of the left subclavian artery (LSA) was totally covered by the stent elephant trunk, and subsequently the LSA root was closed. Afterward, the distal end of the four-branched artificial graft was anastomosed to the proximal end of the stent elephant trunk and aortic wall. Then, systemic perfusion was recovered through the cannulas in the femoral artery, and bleeding was checked in anastomosis sites between the distal end of the four-branched artificial graft and the proximal end of the stent elephant trunk. Finally, the LCCA and LSA were connected to the two 8-mm branches of the four-branched artificial graft, respectively with 5–0 prolene.

#### Step 4

The proximal end of four-branched artificial graft was continuously anastomosed to the artificial graft of step 2 with 4–0 prolene. Afterward, aortic cross clamp was released, and the brachiocephalic trunk reconstruction was performed. Transesophageal echocardiography was conducted before the weaning of CPB to test any abnormality of the valve and the cardiac chambers. All the patients were transferred to the cardiac surgery intensive care unit after the operation.

### Data collection and follow-up

The preoperative data were collected from the medical records, including age, gender, body weight, diabetes mellitus, hypertension, chronic kidney disease, hyperlipidemia, serum creatinine (SCr), smoking, and drinking history. The operation data included operation duration, time of CPB, aortic cross-clamp (ACC), proximal anastomosis, and circulatory arrest (CA). The time of proximal anastomosis was defined as the time interval from the beginning of aortic cross-clamp to the circulatory arrest of the lower part of the body. The data of postoperative complications were also collected, including the drainage volume of the postoperative 24 h, reoperation for bleeding, mechanical ventilation time, hospital stay, paraplegia, stroke, renal failure, gastrointestinal bleeding, and tracheotomy.

Outcomes of 1-year follow-up were obtained by telephone interview, rehospitalization records, and clinical examinations at the outpatient clinic. Telephone interviews were performed at 1, 3, 6, and 12 months. According to the American guidelines, transthoracic echocardiography (TTE) and aortic CTA were performed at 3, 6, and 12 months (4, 5). Aortic root events were defined as follows: aortic root dilation (the diameter of aortic sinus ≥ 45 mm), moderate or greater aortic valve insufficiency.

### Statistical analysis

Continuous variables obeying normal distribution were compared by a *t*-test and expressed as the mean ± standard deviation (*M* ± *SD*). Continuous variables disobeying normal distribution were analyzed by the Mann–Whitney *U*-test and expressed as median (interquartile range). Categorical variables were analyzed by *chi*-square and Fisher exact tests and expressed as percentages. SPSS (IBM SPSS Statistics for Windows, Version 21.0. Armonk, NY: IBM Corp.) was used for the analysis. *P* < 0.05 was considered statistically significant.

## Results

### Preoperative characteristics

The medical record review survey showed that sandwich technique was mainly conducted prior to December 2019, and thereafter, the eversion technique was applied until the end of study. The data of preoperative characteristics are listed in Table 1. There were 64 and 79 patients in the eversion technique group and the sandwich technique group, respectively. There was no significance in the mean age and the percentage of male patients between the eversion technique group and the sandwich technique group (*P* > 0.05, respectively). The preoperative median SCr concentration was 77 μmol/L in the eversion technique group and 91 μmol/L in the sandwich technique group (*P* > 0.05). No statistical differences were observed in hypertension, diabetes mellitus, LVEF, albumin, hyperlipidemia, transient ischemic attack (TIA), cystatin C (Cys-C), D-Dimer, and history of drinking and smoking between two groups (*P* > 0.05, respectively).

**TABLE 1:** Preoperative characteristics.

Categories	Eversion technique (*n* = 64)	Sandwich technique (*n* = 79)	*P*-value
Age, y, *M* ± *SD*	51.3 ± 11.7	50.0 ± 9.0	0.48
Male, *n* (%)	50 (78.1)	59 (74.7)	0.63
BW, kg, *M* ± *SD*	76.1 ± 13.1	78.4 ± 14.1	0.33
Drinking, *n* (%)	22 (34.4)	24 (30.4)	0.61
Smoking, *n* (%)	30 (46.9)	31 (39.2)	0.36
Hypertension, *n* (%)	48 (75.0)	64 (81.0)	0.39
Cerebral disease history, *n* (%)	6 (9.4)	6 (7.6)	0.70
Diabetes mellitus, *n* (%)	2 (3.1)	2 (2.5)	1.00
LVEF < 0.45	2 (3.1)	3 (3.8)	1.00
Albumin, g/L, *M* ± *SD*	36.7 ± 4.2	35.8 ± 4.6	0.22
Hyperlipidemia, *n* (%)	8 (12.5)	9 (11.4)	0.84
Hepatic dysfunction, *n* (%)	10 (15.6)	22 (27.8)	0.081
IIA occlusion, *n* (%)	1 (1.6)	1 (1.3)	1.00
TIA, *n* (%)	2 (3.1)	3 (3.8)	1.00
SCr, μmol/L, median (IQR)	77 (34.5)	91 (68.0)	0.060
Cys-C, mg/L, median (IQR)	0.885 (0.36)	0.94 (0.52)	0.15
D-Dimer, μg/ml, median (IQR)	2.02 (3.00)	2.61 (3.77)	0.52

BW, body weight; IIA, internal iliac artery; TIA, transient ischemic attack; SCr, serum creatinine; Cys-C, cystatin C; IQR, interquartile range.

### Operation data

The operation data are presented in Table 2. The mean operation time in the eversion technique group was shorter than that in the sandwich technique group (466 ± 73 min *vs.* 513 ± 81 min, *P* < 0.001). The average CPB time in the eversion technique group was also shortened compared with that in the sandwich technique group (195 ± 26 min *vs.* 211 ± 40 min, *P* = 0.003). Both proximal anastomosis time and aortic cross-clamp time in the eversion technique group were shown shortened (38 ± 12 min *vs.* 58 ± 20 min, *P* < 0.001; and 120 ± 23 min *vs.* 134 ± 27 min, *P* = 0.002, respectively) compared with sandwich technique group. The proportion of > 600 ml fresh frozen plasma (FFP) in eversion group was reduced than that in sandwich technique group while no statistical differences in packed red blood cells (PRBC) transfusion between the two groups. No patients in both of the two groups underwent aortic root replacement due to the uncontrollable bleeding at the anastomosis.

**TABLE 2:** The operation data.

Categories	Eversion technique (*n* = 64)	Sandwich technique (*n* = 79)	*P*-value
Operation time, min, *M* ± *SD*	466 ± 73	513 ± 81	<0.001
CPB time, min, *M* ± *SD*	195 ± 26	211 ± 40	0.003
ACC time, min, *M* ± *SD*	120 ± 23	134 ± 27	0.002
PA time*, min, *M* ± *SD*	38 ± 12	58 ± 20	<0.001
CAtime, min, *M* ± *SD*	22 ± 8	24 ± 6	0.085
Temperature,^°^C, *M* ± *SD*	26.7 ± 1.1	26.5 ± 1.1	0.33
FFP transfusion, *n* (%)			0.002
≤ 600 ml	57 (89.1)	52 (65.8)	
> 600 ml	7 (10.9)	27 (34.2)	
PRBC transfusion, u, median (IQR)	4 (4)	4 (4)	0.91

CPB, cardiopulmonary bypass; ACC, aortic cross-clamp; PA, proximal anastomosis; CA, circulatory arrest; FFP, fresh frozen plasma; PRBC, packed red blood cell; IQR, interquartile range.

*PA time was defined as the time interval from the beginnings of aortic cross-clamp to circulatory arrest of the lower part of the body.

### Postoperative morbidity

Postoperative morbidity is shown in Table 3. The median drainage volume of the postoperative 24 h was 350 (IQR = 350) ml in the eversion technique group and 300 (IQR = 300) ml in the sandwich technique group (*P* > 0.05). The median time of mechanical ventilation was 37.1 (IQR = 45.9) hours in the eversion technique group and 38.3 (IQR = 43.9) hours in the sandwich technique group (*P* > 0.05). No statistical differences were observed in the incidences of paraplegia (0% *vs.* 1.3%), stroke (6.3% *vs.* 8.9%), renal failure (6.3% *vs.* 6.3%), gastrointestinal bleeding (1.6% *vs.* 1.3%), and tracheotomy (4.7% *vs.* 3.8%) in the eversion technique group and the sandwich technique group.

**TABLE 3:** Postoperative morbidities.

Categories	Eversion technique (*n* = 64)	Sandwich technique (*n* = 79)	*P*-value
First day drainage, ml, median (IQR)	350 (350)	300 (300)	0.13
Reoperation for bleeding, *n* (%)	0 (0)	0 (0)	–
Ventilation, h, median (IQR)	37.1 (45.9)	38.3 (43.9)	0.81
Hospital stay time, d, median (IQR)	18.5 (8)	19 (8)	0.83
Paraplegia, *n* (%)	0 (0)	1 (1.3)	–
Stroke, *n* (%)	4 (6.3)	7 (8.9)	0.79
Gastrointestinal bleeding, *n* (%)	1 (1.6)	1 (1.3)	0.88
Renal failure, *n* (%)	4 (6.3)	5 (6.3)	0.98
Tracheotomy, *n* (%)	3 (4.7)	3 (3.8)	0.79

IQR, interquartile range.

### One-year follow-up outcomes

The outcomes of 1-year follow-up are listed in Table 4. The mortality of the eversion technique group and the sandwich technique group was 6.3% *vs.* 6.3% at 30-day, 9.4% *vs.* 7.6% at 3 months, 9.4% *vs.* 8.9% at 6 months, and 9.4% *vs.* 8.9% at 12 months, respectively (*P* > 0.05). One patient in the eversion technique group and one patient in the sandwich technique group underwent reoperation of aortic root during the follow-up period (*P* > 0.05) due to aortic root event.

**TABLE 4:** One-year follow-up outcomes.

Categories	Eversion technique (*n* = 64)	Sandwich technique (*n* = 79)	*P*-value
Mortality at follow-up, *n* (%)			–
30-day	4 (6.3)	5 (6.3)	0.98
3 months	6 (9.4)	6 (7.6)	0.72
6 months	6 (9.4)	7 (8.9)	0.92
12 months	6 (9.4)	7 (8.9)	0.92
Reoperation at 12 months, *n* (%)			–
TEVAR	2 (3.1)	3 (3.8)	0.83
EVAR	1 (1.6)	0 (0)	–
Aortic root reoperation	1 (1.6)	1 (1.3)	0.88
Aortic arch reoperation	0 (0)	0 (0)	–
Aortic root events at 12 months *n* (%)			–
Aortic root dilation	1 (1.6)	1 (1.3)	0.88
Moderate or severe AI	0	0	–

TEVAR, thoracic endovascular aortic repair; EVAR, endovascular abdominal aortic repair; AI, aortic valvular insufficiency.

## Discussion

The present study aimed to introduce the experiences of adventitial eversion and prosthesis eversion technique in the proximal anastomosis of AAAD. The results demonstrated that patients in the eversion technique group obtained a shorter time in operation, CPB, proximal anastomosis, and aortic cross-clamp compared with the sandwich technique group. Less transfusion of FFP in the eversion technique was needed compared with that in the sandwich group. No statistical differences were found between the two groups in the postoperative morbidities such as the incidences of paraplegia, reoperation for bleeding, stroke, renal failure, gastrointestinal bleeding, and tracheotomy. The 1-year follow-up results showed that the mortality, the incidences of reoperation, and the aortic root event were similar in the two groups.

The International Registry of Acute Aortic Dissection (IRAD) study reported that the early mortality of AAAD surgery had declined from 25 to 18% in the past 17 years (6). In the present study, the 30-day mortality was 6.3% (4/64) in the eversion technique group and 6.3% (5/79, *P* > 0.05) in the sandwich technique group. The early mortality in this study was lower than that in IRAD study. The discrepancy may be due to the difference in patient population. We found that the average age of patients in our study was much younger than that in the IRAD study. In 2011, the first Registry of Aortic Dissection (Sino-RAD) was established in China. The data from Sino-RAD showed that the early mortality of AAAD surgery was 5.3%, which was similar to the early mortality in our study (7).

Over the past decades, several techniques were conducted by the cardiac surgeons to attempt to achieve a safer proximal anastomosis. Prosthesis eversion technique for proximal anastomosis of AAAD was first described by Pretre in 1998 (8). In the subsequent reports, Teflon strip was used to reinforce the proximal anastomosis of the aorta (9, 10). Despite the acceptance of Teflon felt for the proximal anastomosis, limitations have also been reported. Teflon felt could cause extensive adhesions and inflammation, which may prevent the complete healing of the dissected aorta (11, 12). Biological glue was another technique used for the repair of aortic root, which has been shown to improve the results of the AAAD surgery (2, 13). However, the bioglue compound used for hemostasis may increase the risk of aortic root re-dissection and make possible embolisms in cerebral and coronary arteries (14, 15). In the current study, adventitial eversion and prosthesis eversion technique was used for proximal anastomosis with the fact that neither was Teflon strip used to wrap the adventitia of the aortic wall nor was bioglue used to fill the anastomotic sites. During the operation, no intractable bleeding at proximal anastomosis occurred in the eversion technique group, indicating that the anastomosis was intact and satisfactory. The results showed that the combination of adventitial eversion and prosthesis eversion technique had several advantages: (1) the double-layer adventitia was flexible and strong enough to reinforce the aortic wall and control the bleeding from needle holes and small anastomotic tears; (2) the double-layer adventitia was autologous tissue without the reaction of organism on the prosthesis material; (3) the combination of adventitial eversion and prosthesis eversion technique allowed a good vision of the structures for proximal anastomosis, a tension-free and quick proximal anastomosis could be achieved; and (4) the possible bleeding at the proximal anastomosis could be checked and repaired before circulatory arrest by the cardioplegia perfusion in the proximal artificial graft.

Long-term CPB and hypothermia during the surgery are regarded as the risk factors of mortality and morbidity in the aortic arch operation for AAAD (16). Thus, on the premise of safety and fine suture, cardiac surgeons tried different surgical techniques to shorten the time of CPB and hypothermia in AAAD surgery. The time of CPB and hypothermia may be heavily confounded by the extent of tissue destruction. However, we thought that it could also be affected by the choice of anastomosis technique. Our data indicated that the eversion technique group showed a shorter time on operation, CPB, proximal anastomosis, and aortic cross-clamp as well as less application of FFP. It was also reported that the late re-intervention rates of the sandwich technique with Teflon felt and biological glue could reach 23 and 20%, respectively (14, 17–19). During the 1-year follow-up period, only one patient (1/64, 1.6%) in the eversion technique group underwent re-operation of aortic root due to the postoperative aortic root event. Based on the above results, we could conclude that the combination of adventitial eversion and prosthesis eversion technique without Teflon strip or bioglue may be a simple and safe suture method for proximal anastomosis in AAAD patients.

The study also has several limitations. It was a retrospective study from a single center and the sample size was relatively small. A randomized controlled trial of multi-center study with larger sample was needed to confirm the advantages of adventitial eversion and prosthesis eversion techniques. The follow-up period was limited, and long-term follow-up would be carried out in the future study. As the AAAD patient population in China was different from that in Western countries, whether this technique was suitable for the Western population remains to be proved.

## Conclusion

Proximal anastomosis with adventitial eversion and prosthesis eversion technique is a promising surgical option for AAAD patients, with favorable perioperative and 1-year follow-up results.

## Data availability statement

The raw data supporting the conclusions of this article will be made available by the authors, without undue reservation.

## Ethics statement

All the protocols were approved by the Medical Ethics Committee of Qilu Hospital of Shandong University (Reference No. KYLL-2017KS-195). The Ethics Committee waived the requirement of written informed consent for participation.

## Author contributions

CF: writing and correction. SG: data collection. XR, XZ, and CW: data collection and statistical analysis. XP: check and correction. ZM: design and check. KL: design and writing. All authors contributed to the article and approved the submitted version.
